# Acquired L1196M *ALK* mutation in anaplastic lymphoma kinase‐positive anaplastic large cell lymphoma during alectinib administration

**DOI:** 10.1002/jha2.646

**Published:** 2023-01-24

**Authors:** Kazuhiro Noguchi, Yasuhiro Ikawa, Mika Takenaka, Yuta Sakai, Toshihiro Fujiki, Rie Kuroda, Hiroko Ikeda, Takatoshi Abe, Seisho Sakai, Taizo Wada

**Affiliations:** ^1^ Department of Pediatrics, School of Medicine, Institute of Medical Pharmaceutical and Health Sciences, Kanazawa University Kanazawa Japan; ^2^ Department of Diagnostic Pathology Kanazawa University Hospital Kanazawa Japan; ^3^ Department of Pediatric Surgery Kanazawa University Hospital Kanazawa Japan

**Keywords:** alectinib resistance, ALK‐positive anaplastic large cell lymphoma, gatekeeper mutation, L1196M ALK mutation, leukemic presentation

## 1 INTRODUCTION

Targeted therapies such as ALK inhibitors show dramatic responses in various cancers; however, long‐term administration can be associated with acquired drug resistance. Crizotinib is the first ALK inhibitor reported as salvage therapy for refractory anaplastic lymphoma kinase‐positive anaplastic large cell lymphoma (ALK+ALCL). It has been reported to have high complete response (CR) rates (approximately 80%) for paediatric patients with relapsed/refractory ALK+ALCL[1]. However, crizotinib resistance has been reported as a cause of several acquired *ALK* mutations, including the L1196M *ALK* mutation known as the gatekeeper mutation in the ALK kinase domain[2, 3]. To overcome crizotinib resistance, alectinib was developed as a second‐generation ALK inhibitor. It inhibits various crizotinib‐resistant *ALK* mutations, including L1196M[4]. Alectinib‐resistant *ALK* mutations have not been reported in patients with ALK+ALCL, and several patients have been reported to have survived more than 1 year with continuous alectinib administration as well‐tolerated maintenance therapy[5, 6]. Here, we report a paediatric patient with relapsed/refractory ALK+ALCL who acquired L1196M *ALK* mutation and relapsed during alectinib administration.

### 1.1 Case

A 10‐year‐old boy was admitted to our hospital with a fever and post‐auricular swelling. He was subsequently diagnosed with nucleophosmin (*NPM)‐ALK*‐rearranged small cell variant of ALK+ALCL and leukemic presentation, whom we have previously reported[7]. We monitored his serum‐soluble interleukin‐2 receptor (sIL‐2R) levels, the number of tumour cells in the blood using flow cytometric analysis (small‐sized tumour cells; CD30‐5‐8+, large‐sized tumour cells; CD30+), and the expression levels of *NPM‐ALK* mRNA transcripts in the tumour cells by droplet digital polymerase chain reaction (ddPCR) over time as markers of treatment efficacy (Figure S1). Although he showed a refractory treatment course during the ALCL99 chemotherapy protocol, he achieved first CR by alectinib administration and successfully underwent allogeneic bone marrow transplantation (BMT).

Alectinib administration was ceased at the beginning of conditioning therapy in BMT, as previously reported[8]. Engraftment was confirmed 17 days post‐transplantation without *NPM‐ALK* mRNA transcripts in the peripheral blood; however, he relapsed 40 days post‐transplantation with a small cell variant of ALK+ALCL and leukemic presentation. Re‐administration of alectinib resulted in a second CR. Therefore, he continued alectinib administration to maintain remission. Five months after the first relapse, he complained of a high fever and persistent cough. Computed tomography revealed several pulmonary tumours in his right lobe. Pathological examination revealed that the pulmonary tumours were common type ALK+ALCL that consisted of only large‐sized tumour cells despite alectinib administration. At the second relapse, neither tumour cells nor *NPM‐ALK* mRNA transcripts were detected in the blood. He underwent vinblastine administration weekly and successfully achieved a third CR.

To elucidate the mechanism for the relapse despite alectinib administration, RNA sequencing analysis was performed for each of the small‐sized tumour cells purified as CD30 negative population at onset, large‐sized tumour cells purified as CD30 positive population at onset, small‐sized tumour cells at the first relapse and large‐sized tumour cells of pulmonary tumours at the second relapse. The cell separation targeting CD30 was performed as previously reported[7]. RNA sequencing analysis revealed that the large‐sized tumour cells of the pulmonary tumour had acquired L1196M mutation in the *ALK* gene (Table 1). I1461V, K1491R, and D1529E ALK variants, estimated as benign variants in ClinVar, were also detected in all tumour samples from onset to the second relapse. These data indicated that the large‐sized tumour cells that constituted a pulmonary tumour at the second relapse derived from the leukemic lymphoma cells of a small cell variant of ALK+ALCL.

**TABLE 1:** SNPs of the ALK gene of each tumour sample by RNA sequence analysis

Sample	Mutation of *ALK* gene	ClinVar	Nucleotide change	rs number
Small‐sized tumour cells at onset	D1529E	Benign	c.4587C > G	rs1881421
	K1491R	Benign	c.4472A > G	rs1881420
	I1461V	Benign	c.4381A > G	rs1670283
Large‐sized tumour cells at onset	D1529E	Benign	c.4587C > G	rs1881421
	K1491R	Benign	c.4472A > G	rs1881420
	I1461V	Benign	c.4381A > G	rs1670283
Small‐sized tumour cells at the first relapse	D1529E	Benign	c.4587C > G	rs1881421
	K1491R	Benign	c.4472A > G	rs1881420
	I1461V	Benign	c.4381A > G	rs1670283
Large‐sized tumour cells of the pulmonary tumour at the second relapse	**L1196M**	Likely pathogenic	c.3586C > A	rs1057519784
	D1529E	Benign	c.4587C > G	rs1881421
	K1491R	Benign	c.4472A > G	rs1881420
	I1461V	Benign	c.4381A > G	rs1670283

*Note*: Large tumour cells of the pulmonary tumour at the second relapse acquired an L1196M mutation in the *ALK* gene.

Abbreviation: SNPs, single nucleotide polymorphisms.

**FIGURE 1:**
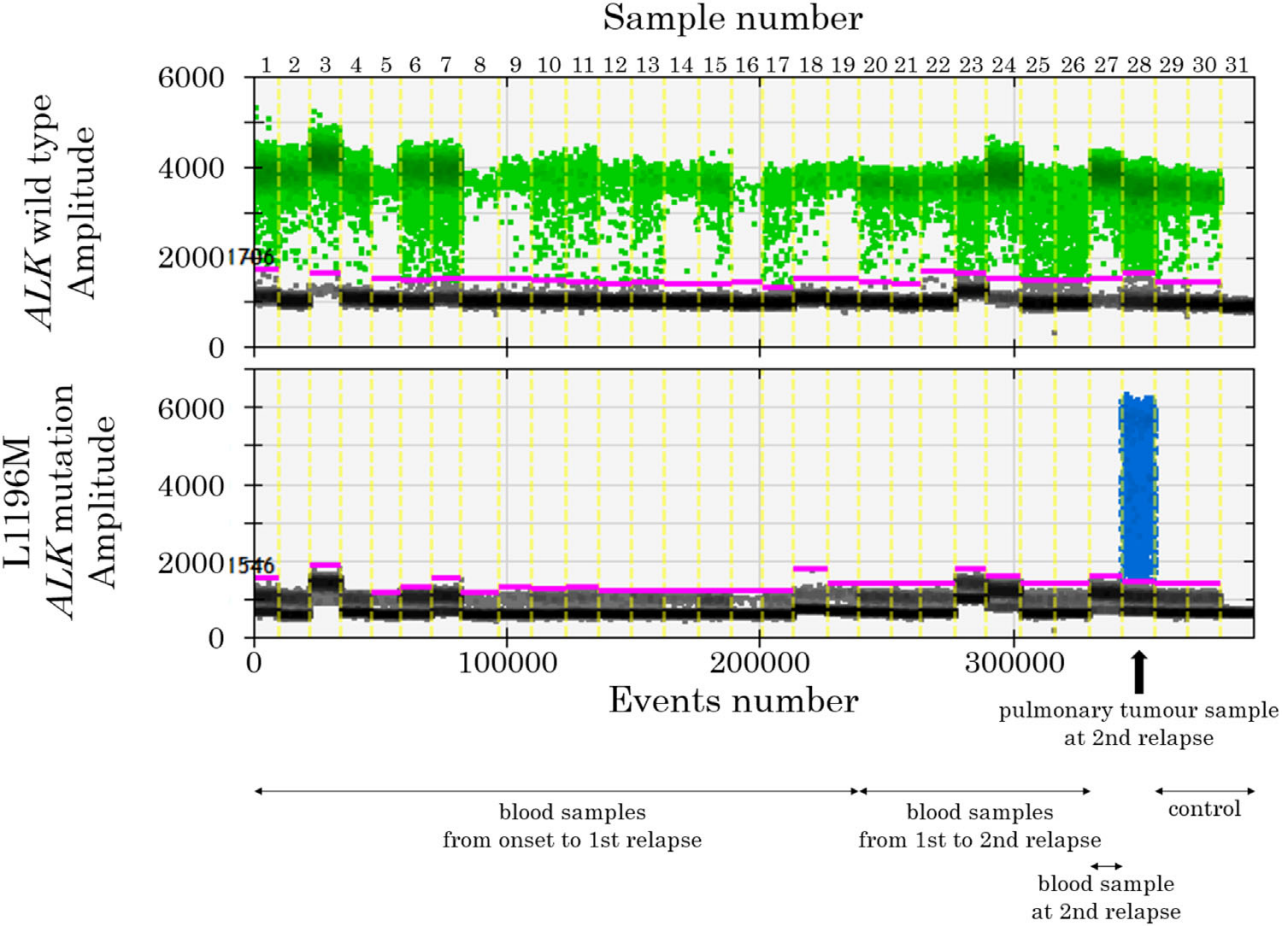
Droplets plot of the digital droplet polymerase chain reaction assay. *ALK* wild‐type positive and negative droplets are presented as green and grey dots, respectively. L1196M *ALK* mutation positive and negative droplets are presented as blue and grey dots, respectively. Thirty‐one samples were analysed. Sample number one is the large‐sized tumour cells purified as the CD30 positive population from the blood sample at onset; sample number two is the small‐sized tumour cells purified as the CD30 negative population from the blood cells at onset; sample numbers three to 19 are the blood samples from onset to the first relapse over time, days 1, 7, 13, 21, 28, 33, 41, 52, 73, 79, 91, 96, 112, 118, 126, 142, and 161 from treatment initiation, respectively; sample numbers 20–26 are blood samples from the first to the second relapse over time, days 189, 205, 215, 219, 221, 243, and 272 from treatment initiation, respectively; sample number 27 is the blood sample at the second relapse; sample number 28 is the pulmonary tumour sample; sample number 29 is the lymph node lesion of another patient with common type anaplastic large cell lymphoma; sample number 30 is a blood sample from a healthy donor; sample number 31 is a negative control (distilled water).

Next, to reveal when L1196M *ALK* mutation emerged in lymphoma cells over time, ddPCR analysis using an L1196M *ALK* mutation detection probe set (LBx Probe ALK L1196M (A071); Riken Genesis, Tokyo, Japan) was performed for each of the blood cell samples preserved over time and the pulmonary lymphoma sample at the second relapse (Figure 1). As a result, L1196M *ALK* mutation was detected only in the pulmonary lymphoma sample and not in any of the blood samples, including the sample at the second relapse. Therefore, we concluded that the acquired L1196M *ALK* mutation induced alectinib resistance.

## 2 DISCUSSION

This is the first report of alectinib resistance in a patient with ALK+ALCL caused by *ALK* mutation. Alectinib has been reported to be effective for crizotinib‐resistant *ALK* mutation, including L1196M gatekeeper mutation, and shows objective responses in crizotinib‐resistant ALK‐rearranged non‐small cell lung cancers (NSCLCs)[4, 9]. While L1196M *ALK* mutation was reported to induce alectinib resistance in a patient with ALK‐rearranged NSCLCs[10]. In a previous study that investigated the susceptibility of *EML4‐ALK* expressing cells with various ALK mutations to each ALK inhibitor, cells with L1196M *ALK* mutation were found to be more susceptible to alectinib than crizotinib but cells with L1196M ALK mutation were less susceptible to alectinib than cells with wild‐type *ALK* [10]. Therefore, reduced alectinib susceptibility by acquired L1196M *ALK* mutation could contribute to a second relapse during alectinib therapy in our patient.

In our patient, the morphology of ALK+ALCL transformed from a small cell variant with leukemic presentation to a common type at the second relapse. Although the mechanisms of morphologic transformation were unclear, morphologic transformation at relapse has been previously reported in a few patients with ALK+ALCL[11, 12]. High *ALK* amplification is known to decrease susceptibility to ALK inhibitors[9]. Our previous study revealed that large‐sized tumour cells expressed ten‐fold more *NPM‐ALK* mRNA transcripts than small‐sized tumour cells, indicating that large‐sized tumour cells acquiring L1196M *ALK* mutation can survive alectinib administration[7].

As continuous alectinib administration may induce *ALK* gatekeeper mutation, including L1196M, we need to establish an exit strategy for alectinib. Moreover, *EML4‐ALK* expressing cells with various *ALK* mutations including L1196M show higher susceptibility to other ALK inhibitors such as ceritinib, brigatinib and lorlatinib, which are off‐label for ALK+ALCL, than alectinib[10]. Therefore, they may become alternative candidates as salvage therapy for refractory ALK+ALCL cases.

## 3 CONCLUSION

L1196M *ALK* mutation, a gatekeeper mutation, was acquired during alectinib administration in ALK+ALCL. The establishment of ‘exit strategies’ for ALK inhibitors is warranted to overcome acquired resistant *ALK* mutation.

## AUTHOR CONTRIBUTIONS

K.N. wrote the manuscript. M.T., Y.S., T.F., R.K., T.A. and S.S. provided care to the patient. H.I. provided pathological diagnosis and analysis. Y.I. was the principal investigator and takes primary responsibility for the paper. T.W. is the chief of our department and provided advice on this study.

## CONFLICT OF INTEREST

The authors have no conflict of interest to declare.

## Supporting information

Supporting Information

## Data Availability

The data that support the findings of this study are available from the corresponding author upon reasonable request.
