# Supplementary material for: Acquired L1196M ALK mutation in anaplastic lymphoma kinase‐positive anaplastic large cell lymphoma during alectinib administration
Source: EJHaem. 2023 Jan 24;4(1):305–8. doi: 10.1002/jha2.646 (PMC9928650; doi:10.1002/jha2.646)
Supplement: Supplementary file 1 — Supporting Information [file JHA2-4-305-s001.docx]

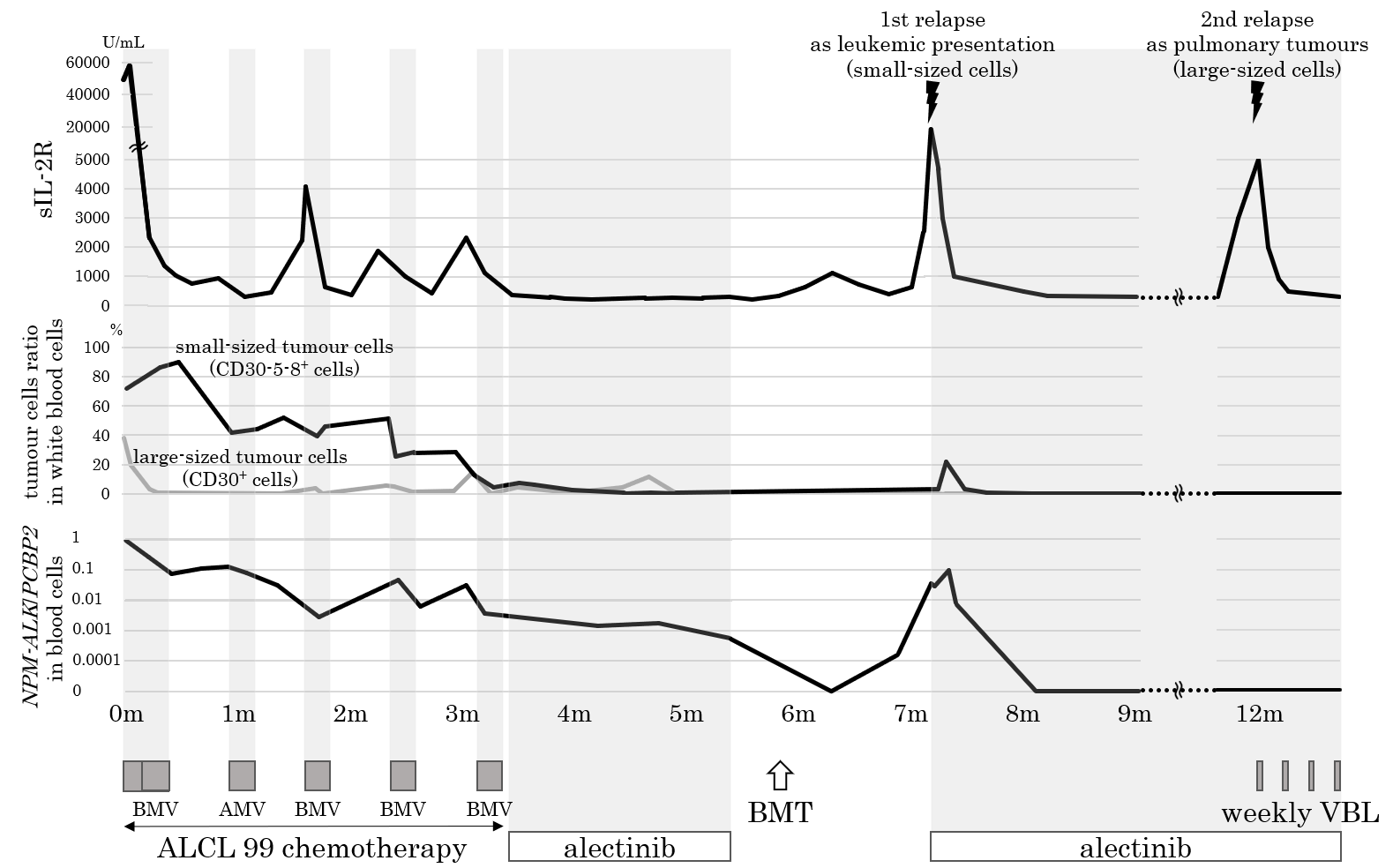


Fig S1. The clinical course with each parameter indicating the residual tumour cells. *NPM-ALK*/*PCBP2* is the ratio of the expression level of the *NPM*-*ALK* fusion gene to the *PCBP2* gene, as indicated with a logarithmic scale. *PCBP2* gene was used as an internal control. sIL-2R, serum-soluble interleukin-2 receptor; NPM, nucleophosmin; ALK, anaplastic lymphoma kinase; PCBP2, poly(rC) binding protein 2; BMT, bone marrow transplantation; VBL, vinblastine.
